# Biomedical Data Sharing and Reuse: Attitudes and Practices of Clinical and Scientific Research Staff

**DOI:** 10.1371/journal.pone.0129506

**Published:** 2015-06-24

**Authors:** Lisa M. Federer, Ya-Ling Lu, Douglas J. Joubert, Judith Welsh, Barbara Brandys

**Affiliations:** NIH Library, Division of Library Services, Office of Research Services, National Institutes of Health, Bethesda, Maryland, United States of America; National Center for Toxicological Research, US Food and Drug Administration, UNITED STATES

## Abstract

**Background:**

Significant efforts are underway within the biomedical research community to encourage sharing and reuse of research data in order to enhance research reproducibility and enable scientific discovery. While some technological challenges do exist, many of the barriers to sharing and reuse are social in nature, arising from researchers’ concerns about and attitudes toward sharing their data. In addition, clinical and basic science researchers face their own unique sets of challenges to sharing data within their communities. This study investigates these differences in experiences with and perceptions about sharing data, as well as barriers to sharing among clinical and basic science researchers.

**Methods:**

Clinical and basic science researchers in the Intramural Research Program at the National Institutes of Health were surveyed about their attitudes toward and experiences with sharing and reusing research data. Of 190 respondents to the survey, the 135 respondents who identified themselves as clinical or basic science researchers were included in this analysis. Odds ratio and Fisher’s exact tests were the primary methods to examine potential relationships between variables. Worst-case scenario sensitivity tests were conducted when necessary.

**Results and Discussion:**

While most respondents considered data sharing and reuse important to their work, they generally rated their expertise as low. Sharing data directly with other researchers was common, but most respondents did not have experience with uploading data to a repository. A number of significant differences exist between the attitudes and practices of clinical and basic science researchers, including their motivations for sharing, their reasons for not sharing, and the amount of work required to prepare their data.

**Conclusions:**

Even within the scope of biomedical research, addressing the unique concerns of diverse research communities is important to encouraging researchers to share and reuse data. Efforts at promoting data sharing and reuse should be aimed at solving not only technological problems, but also addressing researchers’ concerns about sharing their data. Given the varied practices of individual researchers and research communities, standardizing data practices like data citation and repository upload could make sharing and reuse easier.

## Introduction

The importance of sharing and reusing biomedical research data is well established. Sharing data facilitates agile research that allows for quicker translation of research findings into clinical practice [1–3], enhances scientific reproducibility and transparency [4–8], and increases collaboration and interdisciplinary research that helps advance science [9–11]. Collaboration and sharing allow for more effective analysis of the massive datasets that characterize certain data-intensive fields of research, including ‘omics (such as genomics, proteomics, and metabolomics) and population health [12–14]. As the cost of genetic sequencing falls, electronic health records become more widely adopted, and mobile devices incorporate sensors that gather health data from patients, the amount of data available for analysis has exploded [15–18]. Particularly in the setting of rare disease research, sharing data allows researchers to pool several studies in order to increase statistical power and make findings that they could not have achieved individually [19–21].

Funders have also recognized the importance of sharing data and have implemented policies and mandates that encourage researchers to share. Shared data can be repurposed and used in novel ways, thus increasing the return on investment for funded research [22, 23]. Proponents of open science suggest that taxpayers should have access to data arising from federally funded research, a view reflected in the United States Office of Science and Technology Policy’s 2013 memorandum on access to federally funded research results [24]. Accordingly, funders and governmental bodies in the United States, including the National Institutes of Health (NIH) and the National Science Foundation (NSF), and elsewhere, including the Research Councils UK and the European Commission, have instituted policies and issued statements in support of data sharing and openness [25–28].

Despite the many arguments in favor of sharing and open science, researchers often do not share their data. A number of concerns may dissuade researchers from sharing, including concern over other researchers beating the original data collector to publication, fear that others may question the data collector’s findings or conclusions, and worry about people misusing or misinterpreting the data [4, 29]. Practical concerns may also present a roadblock to sharing data; preparing a dataset for sharing can be time-consuming, and researchers are often unaware of repositories available to accept their data [30].

Researchers working with clinical data face their own special set of concerns. Human subject data frequently contain personally identifiable information, and even de-identified data may carry the potential risk of re-identification of subjects [19, 29]. In fact, even when complying with data protection policies such as those prescribed by the Health Insurance Portability and Accountability Act (HIPAA), re-identification of data is a possibility [31]. Obtaining subjects’ consent for sharing datasets can be difficult, particularly since data may end up being used for secondary analysis well after the original study is complete; it is often impossible to foresee what kind of consent might be needed at the time consent is obtained [32]. Electronic health records (EHRs) present a potentially valuable source of clinical data for research, but most systems were designed for clinicians’ ease of use, and frequently lack the kind of structured data that are best suited to sharing and analysis [33].

Sharing basic science research data also presents its own challenges. Data formats change frequently as new technologies and novel experimentation methods arise, making it difficult to coordinate and reuse datasets [34]. Particularly in nascent fields, like proteomics, a lack of standards and formats presents challenges to researchers who would like to share data or collaborate [30]. Working with digital data can be a challenge for researchers who have focused mostly on wet-lab experiments and lack training or a strong background in bioinformatics and computational methods [35].

While concerns over data sharing and reuse are frequently discussed in scientific communities, there are few quantitative studies examining researchers’ attitudes, practices, and perceptions around sharing data. This study aims to better understand the motivations and barriers to data sharing, as well as elucidate differences between the sharing practices of clinical and basic science researchers.

## Methods

### Setting and Population

The NIH Library serves the NIH Intramural Research Program, which is the largest biomedical research program in the world, comprising over 1,200 principal investigators and 4,000 postdoctoral fellows [36]. In addition, the NIH Library serves other NIH employees and staff, as well as customers at related institutions within the Department of Health and Human Services.

The NIH Library launched its Data Services program in October 2013. The program is designed to assist researchers and staff with data management at each step of the research cycle, from conception of the study idea to sharing and archiving of the final research data. To address researchers’ diverse needs, the program includes specialized consultations for research groups, as well as hands-on training in a variety of data-related topics. The survey discussed in this study was conducted during April—May 2014 in order to gain a better understanding of NIH researchers’ data-related training and service needs. The survey sample included a wide variety of respondents in different roles at NIH, including students, fellows, staff scientists, senior scientists, administrators, and other professionals at NIH who collect, utilize, or manage data. However, for the purposes of this paper, only responses from staff scientists and clinical researchers were analyzed.

### Research Instrument

The survey question protocol was tested in a pilot study and revised accordingly. The survey instrument consisted of four parts designed to assess respondents’ attitudes, experience, and knowledge with regard to a variety of data-related topics. This paper reports on the results from sections 2 and 3.

1. **Data Management Tasks**: This section assessed two dimensions of respondents’ experience with specific data management tasks: relevance of the task to their work and their current level of knowledge or expertise with the task. Questions were designed in a pairwise manner, so the first half of the questions addressed the relevance dimension and the second half the expertise dimension of a specific task. Respondents rated each dimension on a 5-point Likert-type scale, from “1—very low” to “5—very high.” Based on feedback from the pilot study that indicated respondents may be so unfamiliar with the tasks that they might not be able to judge relevance and expertise, a non-weighted “not sure” option was also included.
2. **Data Management and Sharing Practices**: This section elicited information about respondents’ experiences with data management and sharing using a nominal scale for dichotomous responses (“yes” or “no”), with related contingency questions.
3. **Data Sharing**: Depending on their responses in section two, respondents were directed to one of two versions of the Data Sharing questions. Respondents who indicated that they had shared data were asked for additional details about their experience with data sharing. Respondents who answered that they had never shared data nor uploaded to a repository were asked to expand upon their reasons for not sharing data.
4. **Demographic Information**: The final section gathered information about respondents’ roles and research at NIH.

The survey was administered using SurveyMonkey, and all responses were anonymous, except when respondents chose to identify themselves as being willing to be contacted for follow-up. To increase the response rate, the survey was publicized through various NIH email lists, including the NIH Library email list and email lists for NIH special interest groups whose members likely work with digital data, such as the Bioinformatics and Biomedical Computing Special Interest Groups. The period for responding to the survey was also extended by several weeks to achieve a higher response rate.

### Analysis Methods

The odds ratio (OR) with corresponding 95% confidence intervals were the primary analyses [37]. Fisher’s exact tests were also used for small samples to avoid effect bias [37]. These two tests examined the potential relationships between variables [37]. When possible, valid responses were aggregated in order to perform OR tests. In the analysis of the Likert-type items, responses such as “not sure” were excluded from the initial analysis because they were not part of the 5-point (i.e., “very low,” “low,” “medium,” “high,” and “very high”) Likert-type scale. However, they were included in the “worst case” sensitivity analyses to estimate the least favorable results. This approach should reduce the impact of excluded data on bias in the results. OR and Fisher’s exact analyses were calculated through two online tools, MedCalc [38] and VassarStats [39], respectively. OR is calculated using a two-by-two contingency table, as demonstrated in Table 1.

**Table 1:** Sample OR contingency table.

	Outcome 1	Outcome 2
**Scientific Staff**	a	c
**Clinical Research Staff**	b	d

For OR tests, p-value was obtained using the z-value calculated from the following formula [37]:

$$SE\left(\text{ln}\left(OR\right)\right)\;=\;\sqrt{\frac{1}{a}+\frac{1}{b}+\frac{1}{c}+\frac{1}{d}}$$

All figures were created with R [40] and RStudio [41] using ggplot2 [42].

### Ethics Statement

The NIH Office of Human Subjects Research Protections within the Office of Intramural Research determined that this survey did not require review by an institutional review board. In lieu of IRB review, the Director, NIH Office of Research Services, approved the survey instrument.

The opening page of the survey noted that survey results could be used for research purposes, but that responses would be anonymized and subjects would not be identified individually. The survey opening page also contained a link to the Library’s Privacy Policy, and contact information for the principal investigator. Although respondents could choose to identify themselves for follow-up, all names and email addresses were removed to anonymize the data before analysis.

## Results and Discussion

### Demographics of Respondents

Of the 190 respondents to the survey, 20 did not select a response for the question about their position and were therefore excluded from analysis. Of the remaining 170 respondents, 113 (67%) identified themselves as Scientific Staff and 22 (13%) identified themselves as Clinical Research Staff, referred to as “scientific” and “clinical” in the tables hereafter. The 35 respondents (21%) who identified themselves as Administrative Staff were excluded from this analysis. Most respondents were NIH employees (68%) or were at NIH on a fellowship appointment (18%) (see Table 2). Because the focus of this study is researchers, only responses from clinical and scientific staff (n = 135) were used for analysis.

**Table 2:** Respondent demographics.

		Position Category			
		Administrative	Clinical	Scientific	Total
**Position Status**	Contractor	5 (2.9%)	0 (0.0%)	13 (7.6%)	18 (10.6%)
	Fellowship Appointment	1 (0.6%)	5 (2.9%)	25 (14.7%)	31 (18.2%)
	Guest Researcher	0 (0.0%)	1 (0.6%)	1 (0.6%)	2 (1.2%)
	NIH Employee	27 (15.9%)	15 (8.8%)	73 (42.9%)	115 (67.6%)
	Summer Student	1 (0.6%)	0 (0.0%)	0 (0.0%)	1 (0.6%)
	Volunteer	1 (0.6%)	1 (0.6%)	1 (0.6%)	3 (1.8%)
	**Total**	35 (20.6%)	22 (12.9%)	113 (66.5%)	170 (100.0%)

### Data Reuse—Relevance and Expertise

**Fig 1:**
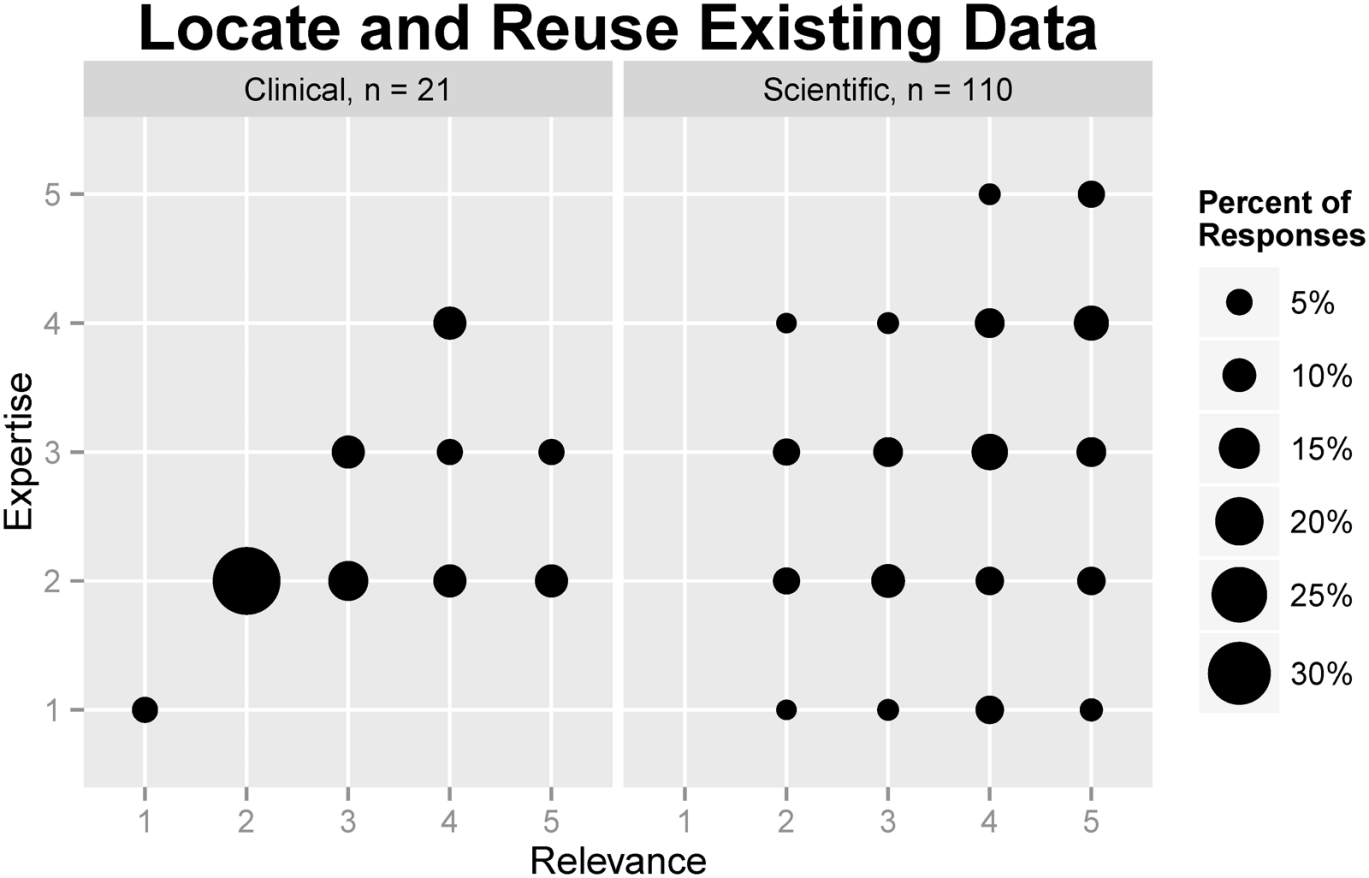
Comparison of self-rated relevance and expertise regarding reusing data among clinical and scientific research staff.

Respondents rated how relevant reusing other researchers’ data was to their work, as well as their current level of expertise in reusing data (see Table 3). A majority of the respondents rated the relevance of finding and reusing datasets as high (31%) or very high (29%). However, nearly three-quarters of respondents considered their expertise very low (11%), low (33%), or medium (29%). Generally, scientific research staff considered the relevance of reusing data higher (median = 4, “high”) than their expertise in doing so (median = 3, “medium”). Clinical staff also rated the relevance of data reuse higher (median = 3, “medium”) than their expertise (median = 2, “low”). Fig 1 demonstrates the relationship between expertise in and relevance of data reuse among scientific and clinical research staff.

**Table 3:** Responses to “Locate and obtain other researchers’ shared data to use in your research, and clean or process it to meet your research needs.”

	Relevance to work						Level of expertise					
	Scientific (n = 113)		Clinical (n = 22)		Total (n = 135)		Scientific (n = 113)		Clinical (n = 22)		Total (n = 135)	
	f	%	f	%	f	%	f	%	f	%	f	%
not sure (0)	2	1.77%	1	4.55%	3	2.22%	2	1.77%	1	4.55%	3	2.22%
very low (1)	0	0.00%	1	4.55%	1	0.74%	14	12.39%	1	4.55%	15	11.11%
low (2)	14	12.39%	7	31.82%	21	15.56%	31	27.43%	14	63.66%	45	33.33%
medium (3)	24	21.23%	5	22.73%	29	21.48%	35	30.97%	4	18.18%	39	28.89%
high (4)	37	32.74%	5	22.73%	42	31.11%	23	20.35%	2	9.09%	25	18.52%
very high (5)	36	31.85%	3	13.64%	39	28.89%	8	7.08%	0	0.00%	8	5.93%

“Not sure” responses (n = 3) were excluded in the initial analysis because they were not part of the 5-point Likert-type scale. The exclusion rates were 2.22% for both the Relevance and Expertise questions.

Next, responses were aggregated to test for differences between the two groups. In considering relevance and expertise, we recoded the 5 ranks of responses into 2 ranks: HIGH (including “medium,” “high,” and “very high” ranks) and LOW (including “low” and “very low” ranks). Odds ratio tests were conducted to test differences in responses for relevance and expertise in data reuse between scientific and clinical respondents.

Results showed that the odds of ranking data reuse as having HIGH relevance in the scientific group are 4.26 times greater than in the clinical group, and the result is statistically significant (OR = 4.26, 95% CI 1.501 to 12.11, p = 0.0065) (see Table 4). In other words, compared with clinical researchers, scientific researchers are more likely to consider data reuse highly relevant to their work. In terms of expertise, the odds of having HIGH expertise ranks in the scientific group are also greater than in the clinical group, and the result is statistically significant (OR = 3.66, 95% CI 1.322 to 10.165, p = 0.0125) (see Table 4).

**Table 4:** Comparison of initial analyses with worst-case scenario analyses.

**Data Reuse Relevance**	**Position**	**HIGH**	**LOW**
"not sure" excluded (OR = 4.2637, 95% CI 1.5012 to 12.1101, p = 0.0065)	Scientific	97	14
	Clinical	13	8
"not sure" included—worst-case scenario (OR = 3.4643, 95% CI 1.2529 to 9.5784, p = 0.0166)	Scientific	97	16
	Clinical	14	8
**Data Reuse Expertise**	**Position**	**HIGH**	**LOW**
"not sure" excluded (OR = 3.6667, 95% CI 1.3225 to 10.1661, p = 0.0125)	Scientific	66	45
	Clinical	6	15
"not sure" included—worst-case scenario (OR = 3.0091, 95% CI 1.1384 to 7.9540, p = 0.0263)	Scientific	66	47
	Clinical	7	15

In order to test if the exclusion of the “not sure” responses biased the results, we inserted these responses back and ran worst-case sensitivity analyses. The worst-case scenario method assumed that the “not sure” responses in the scientific group have the worst possible outcome (LOW) while the “not sure” responses in the clinical group have the best possible outcome (HIGH). The OR results under worse-case scenario were still statistically significant (p<0.05), indicating that the exclusion of the “not sure” responses did not substantially affect our analysis results. Table 4 summarizes the results.

### Uploading to Repositories—Relevance and Expertise

**Fig 2:**
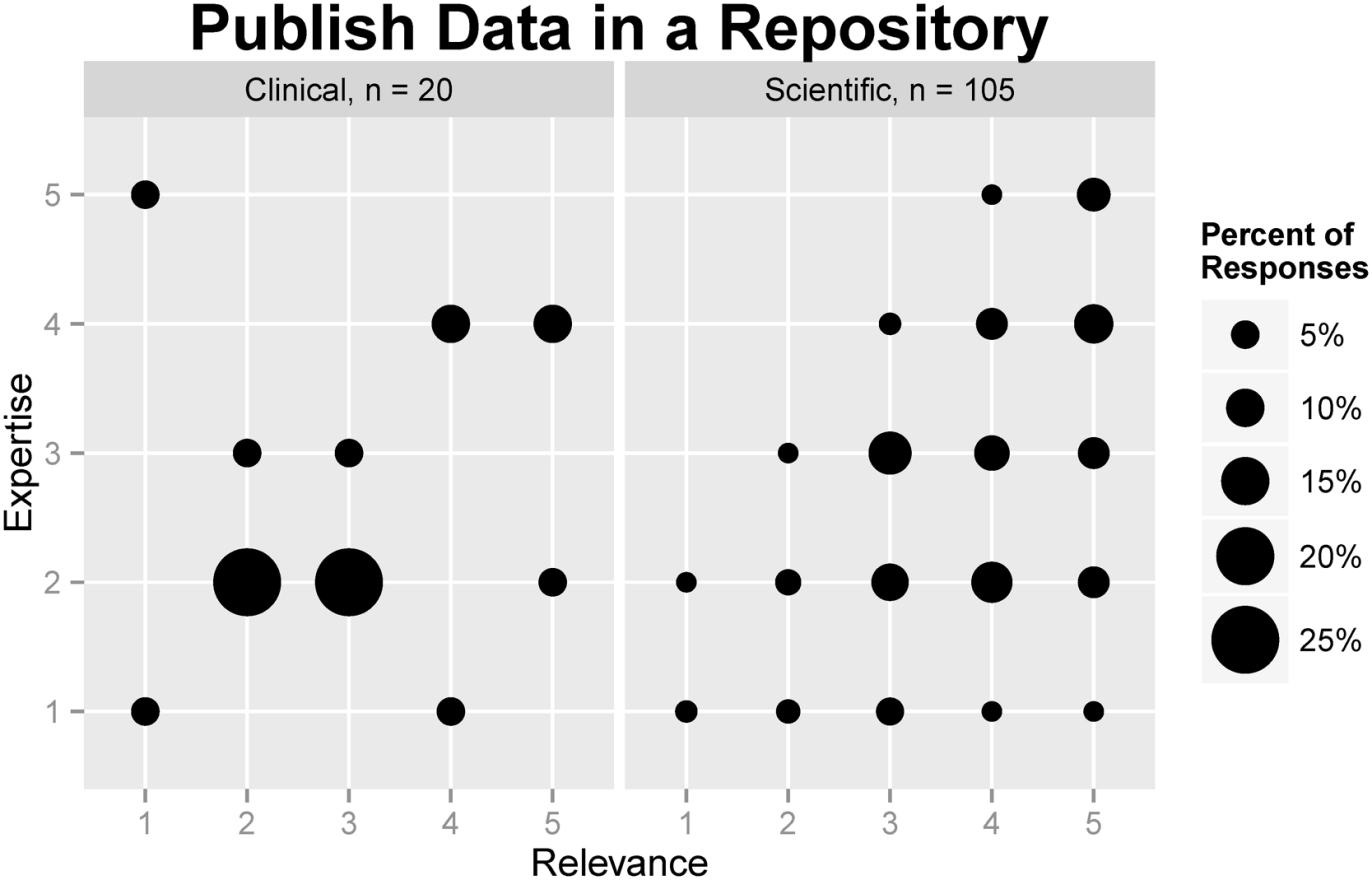
Comparison of self-rated relevance and expertise regarding sharing data in a repository among clinical and scientific research staff.

Respondents also rated relevance and expertise regarding depositing data in a repository (see Table 5). About half of the respondents rated uploading to data repositories as very highly (27%) or highly (24%) relevant to their work, but the majority considered their level of expertise very low (11%), low (34%), or medium (24%). Scientific staff ranked the relevance of sharing data in a repository more highly (median = 4, “high”) than they ranked their expertise in doing so (median = 3, “medium”). Clinical staff also ranked relevance more highly (median = 3, “medium”) than expertise (median = 2, “low”). Fig 2 demonstrates the relationship between expertise in and relevance of repository use among scientific and clinical research staff.

**Table 5:** Responses to “Publish and deposit data in a repository suited to your research field.”

	Relevance to work						Level of expertise					
	Scientific (n = 113)		Clinical (n = 22)		Total (n = 135)		Scientific (n = 113)		Clinical (n = 22)		Total (n = 135)	
	f	%	f	%	f	%	f	%	f	%	f	%
not sure (0)	7	6.19%	2	9.09%	9	6.67%	6	5.31%	1	4.55%	7	5.19%
very low (1)	3	2.65%	2	9.09%	5	3.70%	13	11.50%	2	9.09%	15	11.11%
low (2)	8	7.08%	6	27.27%	14	10.37%	35	30.97%	11	50.00%	46	34.07%
medium (3)	31	27.43%	6	27.27%	37	27.41%	30	26.55%	3	13.64%	33	24.44%
high (4)	30	26.55%	3	13.64%	33	24.43%	20	17.70%	4	18.18%	24	17.78%
very high (5)	34	30.09%	3	13.64%	37	27.41%	9	7.96%	1	4.55%	10	7.41%

Following the same procedures as described above for data reuse, we excluded the “not sure” responses (9 for the Relevance question, and 7 for the Expertise question). The exclusion rates were 6.7% and 5%, respectively. Next, responses were aggregated to test for differences between the two groups. The same re-coding criteria were used: HIGH includes “medium,” “high,” and “very high” ranks; LOW includes “low” and “very low” ranks. Odds ratio results showed that the odds of having HIGH relevance in the scientific group are 5.75 times larger than in the clinical group, and the result is statistically significant (OR = 5.757, 95% CI 1.9341 to 17.1396, p = 0.0017) (see Table 6). This result indicates that scientific researchers are more likely to consider sharing data in a depository relevant to their work. The odds of having HIGH expertise in this task in the scientific group are also greater than in the clinical group (OR = 1.9974), but the result was not significant (95% CI 0.7651 to 5.2146, p = 0.1576) (see Table 6).

**Table 6:** Comparison of initial analyses with worst-case scenario analyses.

**Repository Relevance**	**Position**	**HIGH**	**LOW**
"not sure" excluded (OR = 5.7576, 95% CI 1.9341 to 17.1396, p = 0.0017)	Scientific	95	11
	Clinical	12	8
"not sure" included—worst-case scenario (OR = 3.0159, 95% CI 1.1048 to 8.2327, p = 0.0312)	Scientific	95	18
	Clinical	14	8
**Repository Expertise**	**Position**	**HIGH**	**LOW**
"not sure" excluded (OR = 1.9974, 95% CI 0.7651 to 5.2146, p = 0.1570)	Scientific	59	48
	Clinical	8	13
"not sure" included—worst-case scenario (OR = 1.5782, 95% CI 0.6248 to 3.9864, p = 0.3340)	Scientific	59	54
	Clinical	9	13

Again, we ran the worst-case sensitivity analyses to test if the exclusion of the “not sure” responses biased the results. The worst-case scenario method assumed that the “not sure” responses in the scientific group have the worst possible outcome (LOW) while the “not sure” responses in the clinical group have the best possible outcome (HIGH). The worst-case OR results were consistent with the initial results, with statistical significance in the Relevance question and no statistical significance in the Expertise question. This result indicates that the exclusion of the “not sure” responses did not substantially affect our analysis results. Table 6 summarizes the comparative results.

### Experiences with Sharing Data

Overall, most respondents (61%) reported that they had never uploaded data to a repository (see Table 7). The odds of scientific researchers uploading data to a repository for sharing were somewhat higher than those of the clinical researchers (OR = 1.89), but the result is not statistically significant (95% CI 0.691 to 5.214, p = 0.213).

**Table 7:** Responses to “Have you ever uploaded your data to a public repository?”

	Scientific (n = 113)		Clinical (n = 22)		Total (n = 135)	
	f	%	f	%	f	%
Yes	47	41.59%	6	27.27%	53	39.26%
No	66	58.41%	16	72.73%	82	60.74%

Despite the low levels of sharing in repositories, a majority of respondents (71%) said that they had shared data directly with another researcher (see Table 8). Among scientific staff, almost three-quarters (73%) reported that they had shared data with another researcher, and a majority of clinical research staff (64%) had done so as well. Although there is a 1.5-fold increased odds of sharing data in the scientific group (OR = 1.51, 95% CI: 0.577 to 3.955), this result is not statistically significant (p = 0.399).

**Table 8:** Responses to “Have you ever shared data with another researcher, either informally or through a formal agreement, such as a Material Transfer Agreement or Data Sharing Agreement?”

	Scientific (n = 113)		Clinical (n = 22)		Total (n = 135)	
	f	%	f	%	f	%
Yes	82	72.57%	14	63.64%	96	71.11%
No	31	27.43%	8	36.36%	39	28.89%

### Motivations for Sharing Data

Respondents who indicated that they had previously shared data, either directly with another researcher or by uploading to a repository, were asked about their motivations for doing so. 106 participants provided responses (see Table 9). The most common reason for sharing was to collaborate with a researcher who requested the data (69%). Respondents were also highly motivated by a desire to advance science in a particular area (64%) and to assist a known colleague (49%).

**Table 9:** Responses to “What was your motivation for sharing your data? Please check all that apply.”

	Scientific (n = 93)		Clinical (n = 13)		Total (n = 106)	
	f	%	f	%	f	%
To collaborate with a researcher who requested the data	66	71.73%	7	50%	73	68.87%
To advance science in a particular area	59	63.13%	9	64.28%	68	64.15%
To assist a known colleague	49	53.26%	3	21.42%	52	49.06%
To comply with a requirement to share as a condition of my grant funding or employment	23	25%	3	21.42%	26	24.53%
To assist a junior researcher	23	25%	1	7.14%	24	22.64%
To enhance my professional standing	16	17.39%	2	14.28%	18	16.98%

We used OR tests to analyze whether any of the reasons are associated more with one of the two research groups. For small samples (fewer than 5 responses), Fisher’s exact test was used additionally to avoid bias (see Table 10). None of the results showed any statistical significance (p>0.05). Since the results were not significant, no worst-case sensitivity tests were conducted here to examine the effect of the blank or no responses.

**Table 10:** Odds ratio results for differences between scientific and clinical researchers regarding reasons for sharing.

Reason for data sharing	Position	Yes	No
To collaborate with a researcher who requested the data (OR = 2.0952, 95% CI 0.6446 to 6.8105, p = 0.2188)	Scientific	66	27
	Clinical	7	6
To advance science in a particular area (OR = 0.7712, 95% CI 0.2207 to 2.6950, p = 0.6841)	Scientific	59	34
	Clinical	9	4
To assist a known colleague (OR = 3.7121, 95% CI 0.9595 to 14.3612, p = 0.0574 (Fisher's exact test p = 0.0732))	Scientific	49	44
	Clinical	3	10
To comply with a requirement to share as a condition of my grant funding or employment (OR = 1.0952, 95% CI 0.2773 to 4.3254, p = 0.8967 (Fisher's exact test p = 1))	Scientific	23	70
	Clinical	3	10
To assist a junior researcher (OR = 3.9429, 95% CI 0.4859 to 31.9963, p = 0.1990, (Fisher's exact test p = 0.289))	Scientific	23	70
	Clinical	1	12
To enhance my professional standing (OR = 1.1429, 95% CI 0.2307 to 5.6607, p = 0.8701 (Fisher's exact test p = 1))	Scientific	16	77
	Clinical	2	11

### Sharing Practices

Sharing a dataset alone may not be enough for an outside researcher to be able to understand and reuse the data; additional information, like metadata or a codebook, may be necessary to contextualize and explain the data. Datasets may also need additional preparation to make them useable to other researchers, such as documenting shorthand or abbreviations, adding metadata, or changing formats. Respondents were asked about how much work was required to prepare their datasets and what additional information they supplied to requesters or repositories.

A great deal of variation existed in how much time respondents needed to prepare their data for sharing (see Table 11). Overall, almost a third of respondents (28%) needed more than 10 hours to adequately prepare their data, but a nearly equivalent number (29%) needed no additional time at all, as their data were already ready for sharing. However, none of the clinical research staff responded that their data already existed in a shareable format.

**Table 11:** Responses to “How much time did you or your staff spend preparing your data so it would be ready to share or upload?”

	Scientific (n = 93)		Clinical (n = 13)		Total (n = 106)	
	f	%	f	%	f	%
1–2 hours	15	16.13%	5	38.46%	20	18.87%
3–5 hours	14	15.05%	1	7.69%	15	14.15%
6–10 hours	8	8.60%	2	15.38%	10	9.43%
More than 10 hours	25	26.88%	5	38.46%	30	28.30%
None—my data was already in a form that could be shared	31	33.33%	0	0.00%	31	29.25%
None—my data was not in a form that another researcher would understand, but I made no changes	0	0.00%	0	0.00%	0	0.00%

Most respondents (76 out of 106 people, or 72%) indicated that they had included some additional materials when they shared their data (see Table 12). The most common supplementary material respondents had shared was contextualizing information about the data, such as metadata or a description of the experimental protocol (47%).

**Table 12:** Responses to “Did you provide any additional materials or information besides the dataset?”

	Scientific (n = 93)		Clinical (n = 13)		Total (n = 106)	
Contextualizing information about the data	45	48.39%	5	38.46%	50	47.17%
Codebook explaining variables	29	31.18%	5	38.46%	34	32.08%
Code used with the data, such as R code	24	25.81%	3	23.08%	27	25.47%
Software or program required to access or analyze the data	25	26.88%	1	7.69%	26	24.53%
Nothing—the data required no additional materials to be useful to the requester	24	25.81%	6	46.15%	30	28.30%
Nothing—the data required additional materials to be useful to the requester, but I did not send them	0	0.00%	0	0.00%	0	0.00%

Fisher’s exact tests were conducted through 2 by 2 tables to identify differences regarding supplementary materials that were shared. No significance was found in any of the tables (Fisher’s exact, p >0.1) (see Table 13). In other words, the odds of providing any of the listed supplementary materials did not appear different between the two groups. Although no single type of supplementary information emerged as a more common method for providing documentation, it is encouraging that none of the respondents indicated that they had failed to provide documentation that would be necessary for the requester.

**Table 13:** Scientific group vs. Clinical group: supplementary materials they provided in data sharing.

Shared supplementary materials	Position	Yes	No
Contextualizing information about the data (Fisher’s exact p = 0.5646	Scientific	45	48
	Clinical	5	8
Codebook explaining variables (Fisher’s exact p = 0.7520)	Scientific	29	64
	Clinical	5	8
Code used with the data, such as R code (Fisher’s exact p = 1.0000)	Scientific	24	69
	Clinical	3	10
Software or program required to access or analyze the data (Fisher’s exact p = 0.1793)	Scientific	25	68
	Clinical	1	12
Nothing—the data required no additional materials to be useful to the requester (Fisher’s exact p = 0.1857)	Scientific	24	69
	Clinical	6	7
Nothing—the data required additional materials to be useful to the requester, but I did not send them (Fisher’s exact p = 1.0000)	Scientific	0	93
	Clinical	0	13

### Acknowledgment of Sharing

Respondents who had shared data were asked how they had been acknowledged for contributing their data. Since more than one publication could have arisen from sharing, respondents could select multiple options. 104 participants provided responses. In most cases of data sharing, publication had arisen as a result of the data being shared; only 31% of the respondents said that no publication had yet arisen from the analysis of the shared data (see Table 14).

**Table 14:** Responses to “If another researcher published or presented on results from your shared data, how were you acknowledged?”

	Scientific (n = 91)		Clinical (n = 13)		Total (n = 104)	
Co-authorship	46	50.55%	7	53.85%	53	50.96%
Recognition in the acknowledgement section of the publication	34	37.36%	2	15.38%	36	34.62%
Citation in bibliography	22	24.18%	1	7.69%	23	22.12%
I received no acknowledgement	14	15.38%	2	15.38%	16	15.38%
No publication arose from sharing data	27	29.67%	5	38.46%	32	30.77%

About half of the respondents had been included as a co-author on a publication (51%). The next most common method of noting the contribution of data was recognition in the acknowledgement section of the publication (35%). Several respondents indicated that they had been cited in the bibliography of the publication (22%). However, in a number of cases (15%), respondents reported that they had not been acknowledged for sharing their data.

Fisher’s exact tests were conducted through 2 by 2 tables to identify differences in the ways scientific and clinical researchers were acknowledged. No significance was found in any of the tables (p>0.2). In other words, no significant difference was found between the two groups with regard to any of the listed methods for acknowledging data sharing (see Table 15).

**Table 15:** Scientific group vs. Clinical group: type of acknowledgement of data sharing.

Type of acknowledgement	Position	Yes	No
Co-authorship (Fisher’s exact p = 1.0000)	Scientific	46	45
	Clinical	7	6
Recognition in the acknowledgement section of the publication (Fisher’s exact p = 0.2108)	Scientific	34	57
	Clinical	2	11
Citation in bibliography (Fisher’s exact p = 0.2885)	Scientific	22	69
	Clinical	1	12
I received no acknowledgement (Fisher’s exact p = 1.0000)	Scientific	14	77
	Clinical	2	11
No publication arose from sharing data (Fisher’s exact p = 0.5324)	Scientific	27	64
	Clinical	5	8

### Reasons for Not Sharing Data

Respondents who indicated that they had neither shared data with a researcher nor uploaded to a repository were directed to a question to elicit information about why they had never shared data (see Table 16). Respondents could select more than one of the fifteen possible responses, since multiple reasons might drive their decision not to share. While the list of reasons for not sharing is not completely comprehensive, the fifteen options were based on common reasons for not sharing identified in the existing literature [7, 14, 17, 33]. Twenty participants provided responses.

**Table 16:** Responses to “You have indicated that you have never shared your data nor uploaded to a repository. Please indicate the reason(s) for not sharing your data. Please check all that apply.”

	Scientific (n = 15)		Clinical (n = 5)		Total (n = 20)	
I would be willing to share my data, but I haven't had an opportunity to do so	8	53%	1	20%	9	45%
My data contains personally identifiable information and sharing would compromise my subjects' privacy	2	13%	5	100%	7	35%
I am prohibited from sharing my data for some reason other than subject privacy	2	13%	4	80%	6	30%
I don't know any repositories that accept the kind of data I produce	7	47%	2	40%	9	45%
It's too difficult to prepare my data and documentation for sharing with others	0	0%	0	0%	0	0%
I don't know how to prepare my data and documentation for sharing with others	6	40%	0	0%	6	30%
Repositories' requirements for format or description of data are too difficult to meet	0	0%	0	0%	0	0%
I don't feel I would get credit for sharing my data	1	7%	0	0%	1	5%
I put in a great deal of time and effort to gather my data, and I don't want to give it away	0	0%	1	20%	1	5%
I'm concerned that another researcher could beat me to publication if I share my data	1	7%	0	0%	1	5%
My data has commercial value, so I don't want to give it away for free	0	0%	0	0%	0	0%
I don't think anyone else would have any reason to use my data	4	27%	0	0%	4	20%
It isn't customary to share data in my research field	4	27%	3	60%	7	35%
I'm concerned another researcher might find errors in my data	0	0%	0	0%	0	0%
I'm concerned another researcher might misinterpret my data	1	7%	2	40%	3	15%

Given the small sample sizes (15 vs. 5) in this section and the small values (less than 5) for most of the responses, no inferential statistical tests were conducted here to compare the two groups. However, the top concerns of scientific and clinical researchers seemed different. All of the clinical researchers cited subjects’ privacy as a reason for not sharing, while only two (13%) of the scientific researchers shared this concern. In general, researchers in both categories had diverse reasons for not sharing their data, though many involved a lack of adequate knowledge on how to share data, such as unfamiliarity with existing repositories or data preparation standards.

### Limitations

This study is primarily exploratory in nature and results may not be broadly generalizable. The small size of the sample for this study limits the ability to draw conclusions about the population of NIH researchers as a whole. In particular, clinical researchers were underrepresented. Moreover, the population of NIH researchers may not be representative of the larger biomedical research community on the whole; researchers who work at academic institutions or in the private sector may have different attitudes about sharing data than those who choose a career with a federal agency.

## Conclusions

Sharing research data is a complex issue presenting many challenges that can only be effectively addressed by enlisting the efforts of a variety of stakeholders. While technological barriers to data sharing must be addressed, the scientific community must also evolve in its attitudes and practices to facilitate, encourage, and reward data sharing and reuse. As this study demonstrates, clinical and scientific researchers are not identical in their concerns. Effective methods for encouraging data sharing must take into account the unique needs and challenges of diverse scientific communities.

Though a majority of respondents had shared data with other researchers, or at least indicated they would be willing to do so, fewer researchers had shared data in repositories. Sharing among researchers is a good first step toward increasing access, but systematized methods of sharing may facilitate more widespread access to and reuse of research data. With many different repositories available, including institutional repositories, discipline-specific repositories, and more generalized repositories like Dryad and Figshare, determining where to upload data can be confusing for researchers. Resources like BioMart, a federated search tool that allows users to search across multiple domains at once, and Databib, a curated list of repositories, can help make the task of finding an appropriate repository easier for researchers [34]. Though this study specifically asked about sharing in repositories, new platforms and mechanisms for sharing data merit further exploration. For example, data journals allow authors to publish their data in a way that can be easily cited and may provide ways of sharing data that fit within the framework of more traditional scholarly communication [43]. Improving standards for metadata, provenance, and data publishing is also essential to facilitate sharing and reuse [44].

As this study indicated, many researchers, particularly clinical staff, do not see sharing data in a repository as relevant to their work. Preparing data for sharing in general, and particularly for sharing in a repository, can be a time-consuming process with little payoff for the researcher who is doing the sharing. Funders, institutions, and publishers can all play a role in incentivizing and encouraging data sharing. Many funders, including NIH and NSF, have already begun requiring some grantees to share datasets. A number of publishers also stipulate as a condition of publication that supporting data must be publically available. Institutions can play a role in encouraging sharing by creating policies and providing space for researchers to upload data [45]. Universities can build upon successes with open access policies that encourage or mandate sharing of publications [46].

Clinical researchers’ lower perceived relevance of uploading to a repository may reflect differences in data practices between clinical and basic science research. Because clinical research usually involves human subjects, privacy concerns and regulations may deter clinical researchers from sharing data in repositories. Indeed, among clinical researchers who indicated that they had not shared data, concern for research subjects’ privacy was the most common reason cited in this study. The necessity of de-identifying patient data may also account for the increased likelihood in this study that clinical researchers would need time to prepare their data for sharing. Finally, more specialized or subject-specific repositories exist for basic science research data than for clinical data. For example, of the 57 data repositories listed on NIH’s Data Sharing Repositories website, 37 of them (65%) accept primarily basic science rather than clinical data [47].

As this study demonstrates, little consistency exists with regard to how researchers are acknowledged by those who reuse their shared data. Standardizing a mechanism for data citation could help incentivize sharing by giving researchers credit for their contribution to the scientific community, in much the same way that they receive credit in the form of article citations for their intellectual contributions in the scientific literature. Though a number of respondents in this study indicated they had been co-authors on articles that cited their shared data, co-authorship may not be an appropriate mechanism for acknowledging the contribution of shared data. The International Committee of Medical Journal Editors defines four criteria for authorship: contributing to the design of the work or collection, analysis, or interpretation of data; drafting or significantly revising the work; approving the final draft; and agreeing to be accountable for all questions of integrity or accuracy of the final work [48]. While researchers who share data meet the first criteria, they may not meet the other three, in which case it would be more appropriate to acknowledge their contribution through citation of the dataset, rather than co-authorship. Creating standards for citing datasets is important to ensure that researchers who share data receive credit in ways that appropriately recognize their contribution.

While incentivizing sharing is important, regulatory and policy changes may be needed to remove barriers to sharing and mitigate unintended negative consequences. In addition to creating adequate infrastructure and awareness of outlets for sharing, mechanisms must be created for protecting researchers’ data and ensuring that data are reused responsibly. Particularly with regard to patient data, access to data should be mediated as appropriate for the level of sensitivity of the dataset. Mechanisms like peer review of proposals for reusing research data, data sharing agreements that clearly specify how a dataset may be used, and approval or exemption of data reuse projects by institutional review boards can all help ensure that data are reused with respect for the subjects and the original researchers who gathered the data [7, 49].

Outreach to researchers may help increase awareness about why sharing is important to the biomedical research community, and training and assistance for researchers preparing data for sharing may also be useful. It is essential that the biomedical research community continue to work toward identifying and addressing the challenges that hinder the effective sharing and reuse of research data. This exploratory study has established some possible concerns and perspectives of biomedical researchers, and we hope that it will serve as a foundation for future studies that will further elucidate the barriers to and incentives for sharing within the broader biomedical research community.
